# Transmission Scheduling Schemes of Industrial Wireless Sensors for Heterogeneous Multiple Control Systems

**DOI:** 10.3390/s18124284

**Published:** 2018-12-05

**Authors:** Bongsang Park, Junghyo Nah, Jang-Young Choi, Ick-Jae Yoon, Pangun Park

**Affiliations:** 1 Department of Radio and Information Communications Engineering, Chungnam National University, Daejeon 34134, Korea; bong4ang@cnu.ac.kr; 2 Department of Electrical Engineering, Chungnam National University, Daejeon 34134, Korea; jnah@cnu.ac.kr (J.N.); choi_jy@cnu.ac.kr (J.-Y.C.); ijyoon@cnu.ac.kr (I.-J.Y.)

**Keywords:** transmission scheduling scheme, industrial internet of things, wireless networks, industrial control systems, wireless networked control systems

## Abstract

The transmission scheduling scheme of wireless networks for industrial control systems is a crucial design component since it directly affects the stability of networked control systems. In this paper, we propose a novel transmission scheduling framework to guarantee the stability of heterogeneous multiple control systems over unreliable wireless channels. Based on the explicit control stability conditions, a constrained optimization problem is proposed to maximize the minimum slack of the stability constraint for the heterogeneous control systems. We propose three transmission scheduling schemes, namely centralized stationary random access, distributed random access, and Lyapunov-based scheduling scheme, to solve the constrained optimization problem with a low computation cost. The three proposed transmission scheduling schemes were evaluated on heterogeneous multiple control systems with different link conditions. One interesting finding is that the proposed centralized Lyapunov-based approach provides almost ideal performance in the context of control stability. Furthermore, the distributed random access is still useful for the small number of links since it also reduces the operational overhead without significantly sacrificing the control performance.

## 1. Introduction

Industrial internet of things (IIoT) through wireless sensors and actuators have tremendous potential to improve the efficiency of various industrial control systems in both process automation and factory automation [1,2,3,4,5]. IIoTs are integrated systems of computation, wireless networking, and physical systems, in which embedded devices are connected to sense, actuate, and control the physical plants. In wireless networked control systems (WNCSs), the wireless sensors basically measure the physical plants and transmit its information to the controllers. The controllers then compute the control signal based on the received sensing information in order to manipulate the physical plants through the actuators. The wireless networks of IIoTs provide many benefits such as simple deployment, flexible installation and maintenance, and increased modularity in many practical control systems with the low cost [1].

Unfortunately, the network constraints such as delays and losses can significantly degrade the control performance and can even lead to unstable control systems [1,6]. In the control community, extensive research has been conducted to analyze the communication effects on control systems and design the control algorithm to handle its effects on the control performance [7]. Comparatively, much less work of wireless network design for control systems has been proposed. In fact, current wireless networks do not offer guaranteed stability of heterogeneous multiple control systems over lossy channels [1]. The main reason is that the numerous parameters of the control system and the communication system influence each other due to the complex interactions among different layers. It is important to understand how these communication constraints affect the control stability and performance properties in a quantitative manner. The quantitative result is an important factor to bridge the gap between control and communication layers for the efficient and stable control operations using IIoTs. Furthermore, the communication protocol must guarantee the stability of all control-loops since each industrial process affects the performance of overall connected control systems [7].

The transmission scheduling policies of sensors and controllers must efficiently optimize the traffic generation instance and transmit slot allocation since it directly affects the network delay and loss, and eventually leading to the stability issue of control systems. Decreasing the traffic generation interval of sensors and controllers generally improves the performance of the control system at the cost of lossy and delayed control feedback due to the increasing network congestion. Moreover, increasing the traffic generation rate may not satisfy the schedulability constraint of the communication system. Thus, the transmission scheduling must adjust its operations dependent on the control system requirements and the link conditions.

The main contribution of the paper is to propose three transmission scheduling policies, namely centralized stationary random access, distributed random access, and Lyapunov-based scheduling scheme, of wireless networks to guarantee the stability of heterogeneous multiple control systems over different lossy links. The three transmission scheduling policies are based on the max-min optimization problem where the objective is to maximize the minimum slack of the stability constraint of the control systems. We show the performance of the proposed scheduling schemes in terms of the stability region of heterogeneous multiple control systems over different link conditions.

The paper is organized as follows. Section 2 discusses the related works on both control and communication aspects. In Section 3, we present a general WNCS modeling framework to include both communication constraints such as varying delays, packet losses, and sampling intervals. In Section 4, an illustrative WNCS example is used to present the fundamental performance issues of general communication protocols in terms of the control stability. Based on the fundamental observation, we formulate a novel optimization problem for the transmission scheduling of wireless networks in Section 5. We present three different scheduling schemes, namely centralized stationary random access, distributed random access, and centralized Lyapunov-based schedule schemes, to solve the proposed optimization problem in Section 6, Section 7 and Section 8, respectively. In Section 9, we analyze the robust performance of the proposed three transmission scheduling schemes to guarantee the stability of heterogeneous control systems. Finally, we summarize the contributions of the paper in Section 10.

*Notations:*$Z^{+}$ denotes all nonnegative integers. Normal font *x*, bold font x, and calligraphic font X denote scalar, vector, and set, respectively.

## 2. Related Works

In the control community, extensive research has been conducted to analyze control stability and to design control algorithms by considering the communication constraints [1,7,8]. The control community generally considers network imperfections and constraints such as the varying packet dropouts, network delays, and traffic generation intervals [1]. Note that all these network factors are highly correlated dependent on the assumptions of the NCS literature. However, most NCS studies only consider some of the network effects, while ignoring the other factors, due to the high complexity of the stability analysis. For instance, the network effects of packet dropouts are investigated in [9,10], of time-varying sampling intervals in [11,12], and of delays in [6,13,14]. By considering the structure of NCSs, previous works analyze the stability of control systems by using either only wireless sensor–controller channel (e.g., [15,16]) or both sensor–controller and controller–actuator (e.g., [8,17,18,19]).

In [19], the explicit bounds on the maximum allowable transfer interval and the maximally allowable delay are derived to guarantee the control stability of NCSs, by considering time-varying sampling period and time-varying packet delays. If there are packet losses for the time-triggered sampling, its effect is modeled as a time-varying sampling period from receiver point-of-view. The maximum allowable transfer interval is the upper bound on the transmission interval for which stability can be guaranteed. If the network performance exceeds the given requirements, then the stability of the overall system could not be guaranteed. The developed results lead to tradeoff curves between maximum allowable transfer interval and maximally allowable delay. These tradeoff curves provide effective quantitative information to the network designer when selecting the requirements to guarantee stability and a desirable level of control performance.

In the communication community, most existing approaches design the scheduling algorithms to meet the delay constraint of each packet for a given traffic demand [20,21,22,23]. An interesting design framework is proposed to minimize the energy consumption of the network, while meeting reliability and delay requirements from the control application [24,25]. In [24], modeling of the slotted random access scheme of the IEEE 802.15.4 medium access control (MAC) is developed by using a Markov chain model. The proposed Markov chain model is used to derive the analytical expressions of reliability, delay, and energy consumption. By using this model, an adaptive IEEE 802.15.4 MAC protocol is proposed. The protocol design is based on a constrained optimization problem where the objective function is the energy consumption of the network, subject to constraints on reliability and packet delay. The protocol is implemented and experimentally evaluated on a testbed. Experimental results show that the proposed algorithm satisfies reliability and delay requirements while ensuring a longer network lifetime under both stationary and transient network conditions.

The cross-layer protocol solution, called Breath, is designed for industrial control applications where source nodes attached to the plant must transmit information via multihop routing to a sink [25]. The protocol is based on randomized routing, MAC, and duty-cycling to minimize the energy consumption, while meeting reliability and packet delay constraints. Analytical and experimental results show that Breath meets reliability and delay requirements while exhibiting a nearly uniform distribution of the work load.

Since the joint design of controller and wireless networks necessitates the derivation of the required packet loss probability and packet delay to achieve the desired control cost, we provided the formulation of the control cost function as a function of the sampling period, packet loss probability, and packet delay [26]. We first presented how the wireless network affects the performance of NCSs by showing the feasible region of the control performance. By considering these results, the joint design between communication and control application layers is proposed for multiple control systems over the IEEE 802.15.4 wireless network. In particular, a constrained optimization problem is studied, where the objective function is the energy consumption of the network and the constraints are the packet loss probability and delay, which are derived from the desired control cost. We clearly observe the tradeoff between the control cost and power consumption of the network.

Recently, a novel framework of communication system design is proposed by efficiently abstracting the control system in the form of maximum allowable transmission interval and maximum allowable delay constraints [27,28]. The transmission interval is the traffic generation interval of successfully received information. The objective of the optimization is to minimize the total energy consumption of the network while guaranteeing interval and delay requirements of the control system and schedulability constraints of the wireless communication system. A schedulability constraint is introduced as the sum of the utilization of the nodes, namely the ratio of the delay to the sampling periods. The decision variables are the set of transmission rate and sampling period. The proposed mixed-integer programming problem is converted to an integer programming problem based on the analysis of optimality and relaxation [27]. Then, the centralized resource allocation algorithm gives the suboptimal solution for the specific case of M-ary quadrature amplitude modulation and earliest deadline first scheduling. For a fixed sampling period, the formulation is also extended for any non-decreasing function of the power consumption as the objective function, any modulation scheme, and any scheduling algorithm in [28]. All related works [27,28] propose the centralized algorithm to adapt the communication parameters for the homogeneous control systems over the equal link condition.

In [29], the cross-layer optimized control protocol is proposed to minimize the worst-case performance loss of multiple control systems over a multihop mesh network. The design approach relies on a constrained max–min optimization problem, where the objective is to maximize the minimum resource redundancy of the network and the constraints are the stability of the closed-loop control systems and the schedulability of the communication resources. The stability condition of the control system has been formulated in the form of stochastic transmission interval constraint [19]. The centralized algorithm gives the optimal solutions of the protocol operation in terms of the sampling period, slot scheduling, and routing.

In comparison to these works [27,28,29], the transmission control policies of IIoTs must optimize both traffic generation instance and transmit slot allocations to guarantee the stability of heterogeneous control systems over different link conditions. The earliest deadline first scheduling only guarantees the optimal performance for the homogeneous requirements [30]. Furthermore, the centralized approach generally provides the optimal performance but at the cost of the monitoring and network control overhead and single point failure problem. In this paper, we focus on the robust performance guarantee of the control stability rather than the energy efficiency issue of wireless networks. In fact, researchers have recently applied the IEEE 802.11 standards for the real-time control applications instead of low-power wireless standards such as IEEE 802.15.4 and 802.15.3 [1,31,32].

## 3. System Model

**Figure 1:**
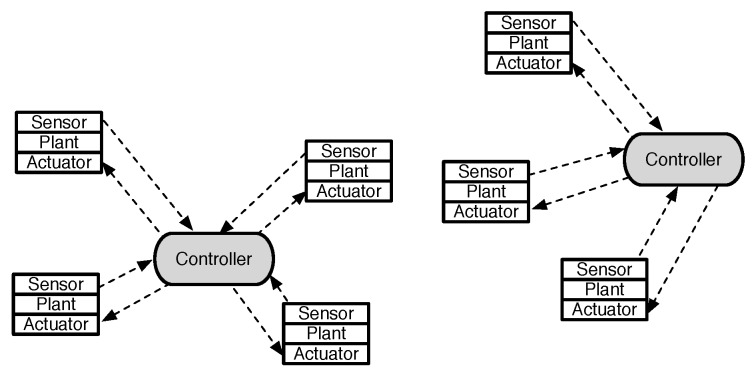
General structure of wireless networked control systems.

Figure 1 illustrates the general structure of multiple control processes over wireless networks. It consists of a number of plants and controllers, which are connected through wireless networks. When the plant and controller are connected over wireless networks, it leads to the following operation aspects of WNCSs in Figure 1.

- While each sensor operates in a time-driven fashion, both the controller and actuator operate in an event-driven fashion. In general, wireless sensors transmit data in each assigned time slot dependent on the transmission scheduling scheme. However, both the controller and actuator only respond to newly received data over unreliable wireless links. We assume that the controllers are collocated with the actuators since the control signal is more critical than the sensing information in many practical NCSs [7].
- In [1], we defined three major metrics of WNCSs, namely sampling interval, packet dropout, and packet delay. Two main reasons of packet dropouts are packet discard due to the control algorithm and packet loss due to the wireless network itself. Most works of control systems model the dropouts as prolongations of the sampling interval [6,19]. The reason is that a new packet is transmitted at the next transmission time with new data if a packet is dropped. Hence, both the controller and actuator observe the time-varying sampling interval even if the sensing and actuating links operate in a fixed time interval. The time-varying sampling interval of successfully received information called transmission interval (TI) effectively captures the essential characteristics of packet dropout and sampling interval [8,19]. The delays are generally assumed to be smaller than the transmission intervals.

The uncertain time-varying TIs and time-varying delays provide the fundamental interactions between control and communication layers [6,19,33]. In the next section, we describe more details of mathematical modelings and assumptions.

### 3.1. Control Aspect

**Figure 2:**
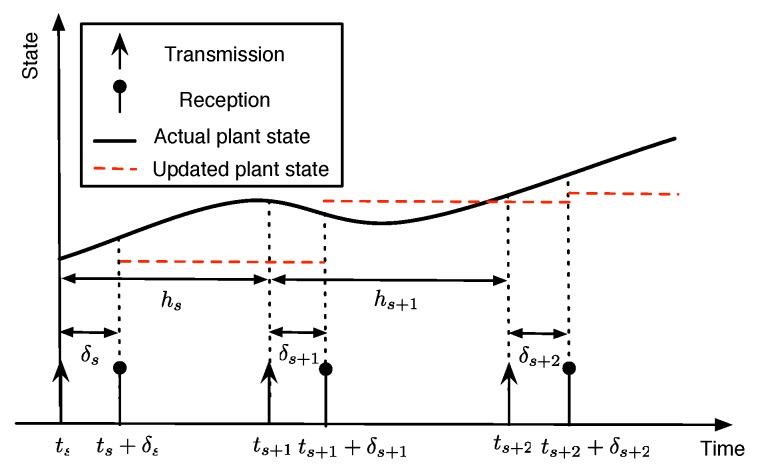
Illustration of a typical evolution of plant state updates at the controller.

Consider a single-hop wireless network consisting with *N* sensor nodes and a controller, as shown in Figure 1. Note that the controller is considered as a base station of a general star network topology. We assume the slotted time with slot index $k\in Z^{+}$. Let us denote the transmission time, $t_{s},s\in Z^{+}$, of *s*th successfully received packet at the controller. At each slot, the transmission scheduling policy determines which of the nodes i∈{1,…,N} can access the network. When the sensor is allowed to transmit, it measures the plant state and sends it over the wireless channel. The packet arrives after the transmission delay $\delta_{s}$ at the controller. Hence, the controller only updates the plant state at time $t_{s}+\delta_{s},\forall s\in Z^{+}$. Figure 2 illustrates the typical evolution of plant state updates at the controller.

The transmission times of successfully received packet satisfy $0\leq t_{0}<t_{1}<t_{2}<\ldots$ and there exists δ>0 such that the TIs $h_{s}=t_{s+1}-t_{s}$ satisfy $\delta \leq t_{s+1}-t_{s}\leq \bar{h}$ for $\forall s\in Z^{+}$, where $\bar{h}$ is the maximum allowable transmission interval (MATI). Furthermore, we assume the maximum allowable delay (MAD) in the sense that $\delta_{s}\in \left[0,\bar{\delta }\right],\forall s\in Z^{+}$, where $0\leq \bar{\delta }\leq \bar{h}$. To guarantee the stability, the TI must satisfy $\delta \leq t_{s+1}-t_{s}\leq \bar{h},s\in Z^{+}$ and the delays satisfy $0\leq \delta_{s}\leq \min\left(\bar{\delta },t_{s+1}-t_{s}\right),\forall s\in Z^{+}$. It implies that each transmitted packet arrives before the next sampling instance. Hence, the delay is smaller than the TI.

In the control community, many studies are conducted to analyze the stability of control systems for a given set of MATI and MAD values [6,19,33]. Hence, it is possible to derive the MATI and MAD requirements as the set of network design parameters by using the stability analysis techniques.

### 3.2. Communication Aspect

Denote the set of *i*’s interfering links, $C_{i}=\left\{j:j\in N\backslash i\right\}$ where N is the total set of nodes for the star topology. Let $u_{i}\left(k\right)=1$ if the node *i* transmits during slot *k*, whereas $u_{i}\left(k\right)$ = 0 otherwise. When $u_{i}\left(k\right)=1$, node *i* generates a new packet and transmits it over the wireless channel to minimize the delay. Hence, the packet delay is fixed to 1 time slot. Note that we motivate the fixed delay between traffic generation instance and transmission schedule through an illustrative example in Section 4. The scheduling constraint is

(1) $$\begin{array}{c} \sum_{i=1}^{N}u_{i}\left(k\right)\leq 1,\;\forall k\in Z^{+} \end{array}$$

It means that the centralized scheduling scheme selects at most one node for transmission at any given time slot *k*. On the other hand, each node decides its transmission for the distributed approach. We assume that the transmission scheduling algorithm is located at the controller for the centralized approach.

Let $d_{i}\left(k\right)$ be the random variable to indicate the successful packet transmission of node *i* to the controller. If node *i* sends a packet at slot *k*, $u_{i}\left(k\right)=1$ and other nodes $j\in C_{j}$ do not transmit, then $d_{i}\left(k\right)=1$ with probability $p_{i}\in \left(0,1\right]$ and $d_{i}\left(k\right)=0$ with probability $1-p_{i}$. If node *i* does not transmit, $u_{i}\left(k\right)=0$, then $d_{i}\left(k\right)=0$ with probability one. We assume the heterogeneous link reliability $p_{i}$ over different links. Hence, the expected delay becomes

(2) $$\begin{array}{c} E\left[d_{i}\left(k\right)\right]=p_{i}E\left[u_{i}\left(k\right)\right]. \end{array}$$

## 4. Fundamental Observation

To analyze the stability of control systems, linear matrix inequality (LMI) conditions were verified on the polytopic overapproximation in [6,19,33]. The LMI conditions were verified using the YALMIP [34] and the SeDuMi solver [35]. We used the analytical technique of the control stability in [33]. This technique effectively analyzes the stability to a given linear time-invariant (LTI) plant model, a LTI controller model, and MATI and MAD bounds on the network uncertainties. In this illustrative example, we first analyzed the batch reactor system [33,36] to demonstrate how stability regions can be visualized. Then, we investigated the fundamental tradeoff between MATI and MAD for the control stability. The network is assumed to incur

- uncertain time-varying TIs $h\in [\underline{h},\bar{h}]$; and
- uncertain time-varying network delays $d\in [\underline{d},\min\left(h,\bar{d}\right)]$.

We fixed $\underline{h}=10$ ms and $\underline{d}=10$ ms due to the slot duration of the typical industrial wireless standards [37].

**Figure 3:**
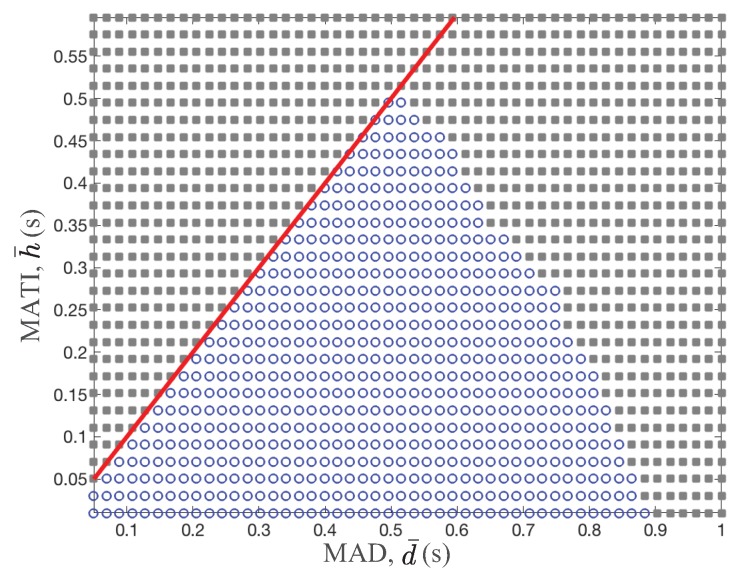
Stability region over different MATI and MAD values. The circle and rectangular marker present the stability and instability operating region of control systems for a given MATI and MAD value.

Figure 3 shows the stability region over different MATI, $\bar{h}=0.01,\ldots ,1$ s and MAD, $\bar{d}=0.01,\ldots ,0.58$ s. The circle and rectangular marker present the stability and instability operating region for a given MATI and MAD value. The solid line represents the assumption of the MATI and MAD constraints, namely $\bar{d}\leq h_{s}\leq \bar{h},\;\forall s\in Z^{+}$. Clearly, as the MATI and MAD values increase, the control system becomes unstable. In other words, the lower are the MATI and MAD values, the better is the control system stability. It is not simple to approximate the boundary between stability and instability region due to the complex stability analysis techniques in Figure 3. However, in general, the MAD requirement of the stability becomes more strict as the MATI requirement is relaxed. Hence, there is a fundamental tradeoff between MATI and MAD requirements for the stability guarantee of control systems.

**Figure 4:**
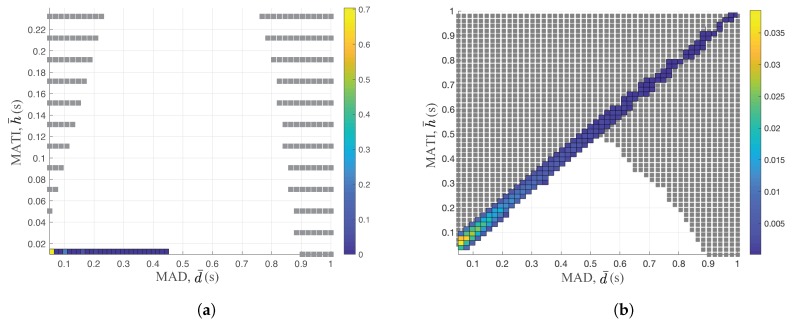
Transmission interval and delay of both TDMA and slotted Aloha schemes over the stability region: (**a**) TDMA performance over stability region; and (**b**) slotted Aloha performance over stability region.

Since the wireless medium is shared between nodes, we analyzed the performance of typical access control schemes in the context of the control stability. In Figure 4, we provide the TI and the delay performance of two well-known schemes, namely time division multiple access (TDMA) and slotted Aloha over the stability region. The color bar shows the probability density function of the TI and the delay measurements of different access schemes. Note that the stability region in Figure 4 is equal to that in Figure 3 with different X and Y scales. The medium access scheme guarantees the control stability if it satisfies the network performance of both TI and the delay inside of the stability region. Hence, the outage probability of the control stability is a good performance metric of wireless networks in the context of the control stability.

Both access schemes rely on the time slotted mode with N=5 and p=0.3. TDMA uses a simple round robin algorithm to assign the slot to each node of the network in Figure 4a. On the other hand, each node randomly decides its transmission based on the traditional slotted Aloha mechanism in Figure 4b. We set the optimal channel access probability of slotted Aloha to maximize the network throughput [38].

By comparing Figure 4a,b, we clearly observed the completely different network performances over the stability region. Since each node of TDMA transmits its corresponding values on the assigned slots, it generates the packet before the transmitting slot. Hence, the delay of the TDMA scheme is constant to 1 time slot, 10 ms, as shown in Figure 4a. On the other hand, the slotted Aloha only generates new packets after it successfully transmits the packets. Hence, the delay of the slotted Aloha is equal to TI. This is the main reason for significantly different behaviors between TDMA and slotted Aloha. In fact, the outage probability of the stability region of slotted Aloha is 0.06 while its corresponding probability is 0 for the TDMA scheme.

The MATI requirement is around 0.89 s when the delay is 10 ms for the TDMA scheme. However, as the MAD constraint is increased to 0.5 s, the MATI requirement becomes around 0.5 s, beyond which the control systems are unstable, as shown in Figure 4b. Increasing the packer delay significantly degrades the stability region. Note that the retransmission of old data to maximize the reliability increases the delay and is generally not useful for control applications [7]. As we increase the MATI requirement, the WNCS becomes more robust since it allows more number of packet losses. Hence, it is great to minimize the time delay between packet generation instance and packet transmission to maximize the MATI requirement and to simplify the protocol operation. This is the main reason of the actual packet transmission right after the packet generation in Section 3.2. Hence, the transmission scheduling policy controls both the packet generation instance and the actual packet transmission in this paper.

## 5. Optimization Problem Formulation

A transmission scheduling policy is an essential component to meet the MATI requirement of node *i* for a given network setup $\left(N,{\bar{h}}_{i},p_{i}\right)$. Our objective was to design low complexity scheduling schemes to optimize the TI performance with respect to the heterogeneous MATI requirements of each node. In this section, we first introduce the performance metric called extended transmission interval (ETI) of the network. Then, we formulate a constrained optimization problem to optimize the ETI performance.

### 5.1. Extended Transmission Interval

Since the exact definition of TI is based on the discrete random event of packet receptions, it is not an efficient performance metric to use for the transmission scheduling policy. Hence, we introduced a continuous version of the TI metric, called the ETI metric. The extended transmission interval describes how old the information is from the controller perspective.

**Figure 5:**
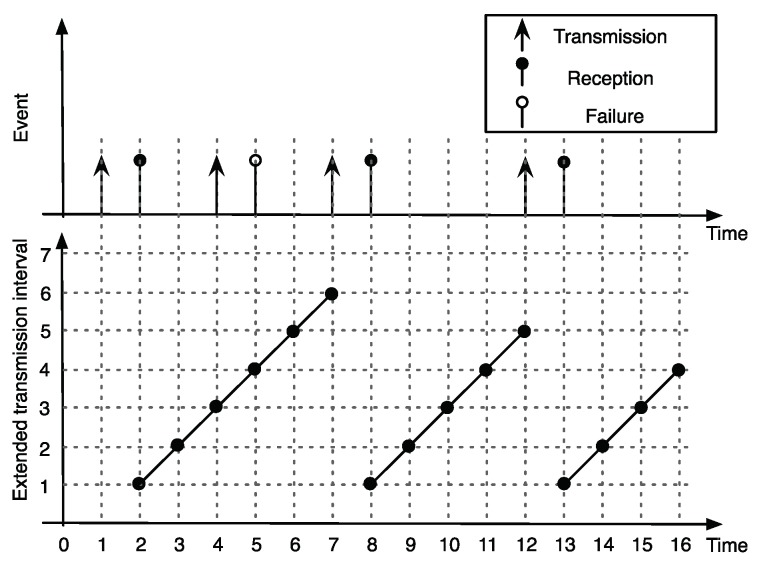
Illustration of a typical evolution of ETI.

Figure 5 illustrates the evolution of ETI for a given sequence of packet deliveries of node *i*. Let $\tau_{i}\left(k\right)$ denote the positive integer to represent the ETI value of node *i* at slot *k*. We reset $\tau_{i}\left(k+1\right)=1$ if the controller receives a packet from node *i* at slot *k*. As a reminder, the received packet was generated at the beginning of slot *k*. However, if the controller does not receive a packet, then we increase $\tau_{i}\left(k+1\right)=\tau_{i}\left(k\right)+1$. Hence, the iterative update of $\tau_{i}\left(k\right)$ is

(3) $$\begin{array}{c} \tau_{i}\left(k+1\right)=\left\{\begin{array}{ll} 1 & \;\mathrm{if}\;d_{i}\left(k\right)=1\\ \tau_{i}\left(k\right)+1 & \;\mathrm{otherwise} \end{array}\right. \end{array}$$

Note that the ETI linearly increases when there is no packet reception, as shown in Figure 5.

The ETI constraint of each link is

(4) $$\begin{array}{c} \lim_{K\to \infty }\frac{1}{K}\sum_{k=1}^{K}E\left[\tau_{i}\left(k\right)\right]\leq {\bar{h}}_{i}\;\forall i\in \left\{1,\ldots ,N\right\} \end{array}$$

Since we are interested on the robust ETI performance with respect to the MATI requirement of each node, we define the ETI slack of node *i* as ${\bar{h}}_{i}-\tau_{i}\left(k\right)$ at slot *k*. Hence, the expected value of the ETI slack of node *i* is

(5) $$\begin{array}{c} \lim_{K\to \infty }\frac{1}{K}E\left[\sum_{k=1}^{K}{\bar{h}}_{i}-\tau_{i}\left(k\right)\right]\;\forall i\in \left\{1,\ldots ,N\right\} \end{array}$$

### 5.2. Optimization Problem

By considering the ETI metric with the robustness criterion, our objective is to maximize the minimum ETI slack of the network. We formulate the constrained optimization problem

(6a) $$\begin{array}{c} \min\;\eta \; \end{array}$$

(6b) $$\begin{array}{c} s.t.\;\lim_{K\to \infty }\frac{1}{K}E\left[\sum_{k=1}^{K}\tau_{i}\left(k\right)-{\bar{h}}_{i}\right]\leq \eta \;\forall i\in \left\{1,\ldots ,N\right\} \end{array}$$

(6c) $$\begin{array}{c} \sum_{i=1}^{N}u_{i}\left(k\right)\leq 1\;\forall k\in Z^{+}\; \end{array}$$

Equations (6b) and (6c) present the minimum ETI slack value constraint and the schedulability constraint, respectively. The optimal solution of the optimization problem assigns more network resources as ${\bar{h}}_{i}$ decreases, i.e., more network resources for faster control systems.

In the following section, we propose three low-complex scheduling schemes based on the optimization problem in Equation (6). Three transmission scheduling policies are centralized random access scheme, distributed random access scheme, and centralized Lyapunov-based scheduling scheme. The first two approaches are randomized methods and the third one is a deterministic approach in order to assign the slot resources to all requirement sets ${\bar{h}}_{i},\forall i\in \left\{1,\ldots ,N\right\}$.

## 6. Centralized Random Access Scheme

In this section, we propose the ETI optimization problem for the stationary randomized scheduling scheme in a centralized manner. The controller selects node *i* with probability $\alpha_{i}\in \left(0,1\right]$ in each time slot. Hence, the randomized scheduling scheme is the vector of scheduling probabilities $\alpha =(\alpha_{1},\ldots ,\alpha_{N})$. They select nodes randomly based on the fixed scheduling probabilities α.

Since we consider the stationary random scheduling, we first derive the expected behavior of ETI in the following proposition.

**Proposition** **1.** *The long-term expected ETI of node i is*

(7) $$\begin{array}{c} \lim_{K\to \infty }\frac{1}{K}\sum_{k=1}^{K}E\left[\tau_{i}\left(k\right)\right]=\frac{1}{p_{i}\alpha_{i}} \end{array}$$

**Proof.**  The proof of Proposition 1 is provided in Appendix A. □

By using Proposition 1 and the schedulability constraint of α, the optimization problem in Equation (6) is reformulated as

(8a) $$\begin{array}{c} \min_{\alpha ,\eta }\;\eta \; \end{array}$$

(8b) $$\begin{array}{c} s.t.\;\frac{1}{p_{i}\alpha_{i}}-{\bar{h}}_{i}\leq \eta \;\forall i\in \left\{1,\ldots ,N\right\} \end{array}$$

(8c) $$\begin{array}{c} \sum_{i=1}^{N}\alpha_{i}\leq 1\; \end{array}$$

Note that Equation (8c) represents the schedulability constraint.

### Optimal Solution

Since the proposed optimization problem in Equation (8) is a convex problem, we obtain the optimal stationary scheduling probabilities $\alpha^{*}$ by analyzing the KKT conditions of the problem. The Lagrange function of the problem is

(9) $$\begin{array}{c} L\left(\alpha ,\eta ,\lambda ,\gamma \right)=\eta +\sum_{i=1}^{N}\lambda_{i}\left(\frac{1}{p_{i}\alpha_{i}}-{\bar{h}}_{i}-\eta \right)+\gamma \left(\sum_{i=1}^{N}\alpha_{i}-1\right) \end{array}$$

where $\lambda =(\lambda_{1},\ldots ,\lambda_{N}),\lambda \geq 0$ and γ are the Lagrange multipliers due to Equations (8b) and (8c), respectively.

The KKT conditions are

- Stationarity with $\alpha_{i}$
  (10) $$\begin{array}{c} \nabla_{\alpha_{i}}L\left(\eta ,\alpha_{i},\lambda_{i},\gamma \right)=0 \end{array}$$
- Stationarity with η
  (11) $$\begin{array}{c} \nabla_{\eta }L\left(\eta ,\alpha_{i},\lambda_{i},\gamma \right)=0 \end{array}$$
- Complementary slackness of Equation (8b)
  (12) $$\begin{array}{c} \lambda_{i}\left(\frac{1}{p_{i}\alpha_{i}}-{\bar{h}}_{i}-\eta \right)=0 \end{array}$$
- Complementary slackness of Equation (8c)
  (13) $$\begin{array}{c} \gamma \left(\sum_{i=1}^{N}\alpha_{i}-1\right)=0 \end{array}$$
- Primal feasibility
  (14) $$\begin{array}{c} \frac{1}{p_{i}\alpha_{i}}-{\bar{h}}_{i}-\eta \leq 0 \end{array}$$
  (15) $$\begin{array}{c} \sum_{i=1}^{N}\alpha_{i}\leq 1\; \end{array}$$
- Dual feasibility
  (16) $$\begin{array}{c} \lambda_{i}\geq 0,\gamma \geq 0 \end{array}$$

The first stationarity condition, $\nabla_{\alpha_{i}}L\left(\alpha_{i},\lambda_{i},\gamma \right)=0$, gives

(17) $$\begin{array}{c} \frac{\lambda_{i}}{p_{i}\alpha_{i}^{2}}=\gamma \end{array}$$

from the partial derivation. With a similar method, the second stationarity condition, $\nabla_{\eta }L\left(\eta ,\alpha_{i},\lambda_{i},\gamma \right)=0$, gives

(18) $$\begin{array}{c} \sum_{i=1}^{N}\lambda_{i}=1\;. \end{array}$$

We obtain that either γ=0 or $\sum_{i=1}^{N}\alpha_{i}=1$ from the complementary slackness of Equation (13). However, Equation (17) implies γ>0 since the value of γ is zero if $\lambda_{i}=0$ or $\alpha_{i}\to \infty$, which conflicts the conditions of Equation (18) or $\alpha_{i}\in \left(0,1\right]$, respectively. Hence, we obtain

(19) $$\begin{array}{c} \sum_{i=1}^{N}\alpha_{i}=1 \end{array}$$

since γ>0. We separate node *i* into two groups, namely $\lambda_{i}=0$ and $\lambda_{i}>0$, based on the dual feasibility $\lambda_{i}\geq 0$.

If node *i* has $\lambda_{i}=0$, then $\alpha_{i}=0$ from Equation (17) since γ>0. On the other hand, if node *i* has $\lambda_{i}>0$, then

(20) $$\begin{array}{c} \alpha_{i}=\frac{1}{\left({\bar{h}}_{i}+\eta \right)p_{i}} \end{array}$$

due to the complementary slackness of Equation (12). For any fixed value of γ>0, the randomized scheduling probability of node *i* is given by Equation (20).

Our objective is to find the optimal values to meet the constraints of Equations (19) and (20). In Equation (20), η is only unknown variable to compute the scheduling probability. Hence, we first find the optimal value of $\eta^{*}$ satisfying Equations (19) and (20). Now, let us derive the boundaries of η. The minimum value of η is $1/p_{i}-{\bar{h}}_{i}$ if $\alpha_{i}=1$ from Equation (20). Hence, the feasible range of η is

(21) $$\begin{array}{c} \frac{1}{p_{i}}-{\bar{h}}_{i}\leq \eta \leq 0\;. \end{array}$$

From Equation (20), a decreasing value of η, the probability $\alpha_{i}$ increases. As η converges to the lower bound, $\eta \to 1/p_{i}-{\bar{h}}_{i}$, Equation (19) becomes $\sum_{i=1}^{N}\alpha_{i}\geq 1$. On the other hand, as η→0, then $\sum_{i=1}^{N}\alpha_{i}=\sum_{i=1}^{N}\frac{1}{{\bar{h}}_{i}p_{i}}\ll 1$ due to Equation (20). Hence, there is a unique value of $\eta^{*}$ to meet Equation (19) due to the monotonicity of $\alpha_{i}$ with respect to η. By gradually increasing η from $1/p_{i}-{\bar{h}}_{i}$ and adjusting $\alpha_{i}$ using Equation (20), we find the unique set of $\alpha^{*}$ and $\eta^{*}$ that satisfies Equations (19) and (20).

Next, we compute the optimal set of $\lambda^{*}$ and $\gamma^{*}$. With a similar method, the boundaries of γ are

(22) $$\begin{array}{c} 0\leq \gamma \leq \bar{\gamma }=\max_{i\in \{1,\ldots ,N\}}\frac{1}{p_{i}\alpha_{i}^{2}} \end{array}$$

The upper bound of γ is derived from Equations (17) and (19). By gradually decreasing γ from $\bar{\gamma }$, we adjust $\lambda_{i}=\gamma p_{i}\alpha_{i}^{2}$ from Equation (17). We then obtain the optimal set of $\lambda^{*}$ and $\gamma^{*}$ for Equation (18). Note that the unique vector $\left(\alpha^{*},\eta^{*},\lambda^{*},\gamma^{*}\right)$ fulfills the KKT conditions.

## 7. Distributed Random Access Scheme

In this section, we present a distributed random access where each node decides its transmission probability so that the minimum ETI slack of the network is maximized. Let us assume that each node *i* transmits a packet with probability $\beta_{i}$ in each slot. As a reminder, the set of *i*’s interfering links is $C_{i}$. Let $\beta =(\beta_{1},\ldots ,\beta_{N})$ be the vector of transmission probabilities of all nodes. Hence, a transmission of node i∈{1,…,N} is successful if and only if no node in $C_{i}$ transmits during the transmission of node *i*. Hence, the successful transmission probability of node *i* is

(23) $$\begin{array}{c} p_{i}\beta_{i}\prod_{j\in C_{i}}\left(1-\beta_{j}\right) \end{array}$$

By applying Equation (23) to Proposition 1, the long-term expected ETI of node *i* is

(24) $$\begin{array}{c} \lim_{K\to \infty }\frac{1}{K}\sum_{k=1}^{K}E\left[\tau_{i}\left(k\right)\right]=\frac{1}{p_{i}\beta_{i}\prod_{j\in C_{i}}\left(1-\beta_{j}\right)} \end{array}$$

After substituting Equation (24) into Equation (4) of the ETI constraint, we obtain

(25) $$\begin{array}{c} \frac{1}{p_{i}{\bar{h}}_{i}\beta_{i}\prod_{j\in C_{i}}\left(1-\beta_{j}\right)}\leq 1\;. \end{array}$$

Motivating by the network utility problem [39,40], we formulate a constrained optimization problem to optimize the transmission probability of nodes. Our objective is to maximize the minimum ETI slack with respect to ${\bar{h}}_{i}$ of the network. After some manipulations of Equation (6), we propose the max-min robust optimization problem

(26a) $$\begin{array}{c} \max_{\beta ,\psi }\;\psi \; \end{array}$$

(26b) $$\begin{array}{c} s.t.\;a_{i}\beta_{i}\prod_{j\in C_{i}}\left(1-\beta_{j}\right)\geq \psi \;\forall i\in \left\{1,\ldots ,N\right\} \end{array}$$

where $a_{i}=p_{i}{\bar{h}}_{i}$. Notice that ψ>1 is necessary for the feasibility of ETI constraint of Equation (4). Different fairness notions corresponding to different utility functions are discussed in [39,40].

Unfortunately, the proposed max-min robust problem in Equation (26) is a non-convex problem. Next, we convert the problem in Equation (26) to a convex optimization problem in order to design the distributed random access scheme.

**Proposition** **2.** *The max-min robust problem in Equation* (26) *is equivalent to the following convex programming problem*

(27a) $$\begin{array}{c} \min\;\frac{1}{2}\sum_{i=1}^{N}\nu_{i}^{2}\; \end{array}$$

(27b) $$\begin{array}{c} s.t.\;\nu_{i}\leq \log a_{i}+\log\beta_{i}+\sum_{j\in C_{i}}\log\left(1-\beta_{j}\right) \end{array}$$

(27c) $$\begin{array}{c} \;\nu_{i}=\nu_{j}\;\forall i\in \left\{1,\ldots ,N\right\},\forall j\in C_{i} \end{array}$$

**Proof.**  The proof of Proposition 2 is provided in Appendix B. □

### Optimal Solution

A distributed algorithm can effectively obtain the optimal access probability of the previous convex optimization problem in Equation (27). In this section, we show how globally optimal access probability is obtained in a distributed manner. When we replace the equality constraints $\nu_{i}=\nu_{j}$ of Equation (27) by two inequality constraints, $\nu_{i}\leq \nu_{j}$ and $\nu_{i}\geq \nu_{j}$, the Lagrange function of the problem is

(28) $$\begin{array}{c} L(\beta ,\nu ,\mu ,\omega )=\frac{1}{2}\sum_{i\in N}\nu_{i}^{2}+\sum_{i\in N}\mu_{i}\left(\nu_{i}-\log a_{i}-\log\beta_{i}-\sum_{j\in C_{i}}\log\left(1-\beta_{j}\right)\right)+\sum_{i\in \{1,\ldots ,N\}}\sum_{j\in C_{j}}\omega_{i,j}\left(\nu_{i}-\nu_{j}\right) \end{array}$$

where $\mu =(\mu_{1},\ldots ,\mu_{N})$ and $\omega =(\omega_{i,j},i\in \left\{1,\ldots ,N\right\},j\in C_{i})$ are the Lagrange multipliers and $\nu =(\nu_{1},\ldots ,\nu_{N})$.

Our basic idea is to apply the gradient project method for the dual problem $\max_{\beta ,\nu }D\left(\beta ,\nu \right)$ where the dual function is $D\left(\beta ,\nu \right)=\min_{\beta ,\nu }L\left(\beta ,\nu ,\mu ,\omega \right)$ [41]. By considering the stationarity condition of the Lagrange function, they give $\frac{\partial L}{\partial \beta_{i}}=0$

(29) $$\begin{array}{c} \beta_{i}=\frac{\mu_{i}}{\mu_{i}+\sum_{j\in C_{i}}\mu_{j}} \end{array}$$

and $\frac{\partial L}{\partial \nu_{i}}=\nu_{i}+\mu_{i}+\sum_{j\in C_{i}}\omega_{i,j}-\omega_{j,i}=0$

(30) $$\begin{array}{c} u_{i}={\left[-\lambda_{i}-\sum_{j\in L_{i}}\left(v_{i,j}-v_{j,i}\right)\right]}^{-} \end{array}$$

where ${\left[z\right]}^{-}=\min\left(z,0\right)$. Note that $\beta_{i}$ satisfies the constraints $0\leq \beta_{i}\leq 1$. The Lagrange multipliers of the gradient project method are adjusted in the direction of the gradient ∇D(β,ν):

(31) $$\begin{array}{c} \mu_{i}\left(n+1\right)={\left[\mu_{i}\left(n\right)+\epsilon_{n}\frac{\partial D}{\partial \mu_{i}}\right]}^{+} \end{array}$$

(32) $$\begin{array}{c} \omega_{i,j}\left(n+1\right)={\left[\omega_{i,j}\left(n\right)+\epsilon_{n}\frac{\partial D}{\partial \omega_{i,j}}\right]}^{+} \end{array}$$

Here, $\epsilon_{n}>0$ is the step size at the *n*th iteration, and ${\left[z\right]}^{+}=\max\left(z,0\right)$. The gradient are $\frac{\partial D}{\partial \mu_{i}}=\nu_{i}-\log a_{i}-\log\beta_{i}-\sum_{j\in C_{i}}\log\left(1-\beta_{j}\right)$ and $\frac{\partial D}{\partial \omega_{i,j}}=\nu_{i}-\nu_{j}$.

## 8. Centralized Lyapunov-Based Scheduling Scheme

The Lyapunov optimization theory is extensively applied to the general communication and queueing systems [42]. Using Lyapunov optimization techniques, we derive the centralized scheduling scheme for the ETI optimization problem in Equation (6). The Lyapunov-based scheduling scheme uses the feedback ETI state of nodes in order to reduce the value of the Lyapunov function. We define the Lyapunov function to give a large positive scalar when nodes have high ETI with respect to the MATI ${\bar{h}}_{i}$. Hence, the Lyapunov-based scheduling approach basically tries to minimize the growth of its function.

Next, we define the fundamental components of the Lyapunov-based scheduling scheme, namely Lyapunov function with notions of the ETI debt. Let us denote $x_{i}\left(k\right)$ as the ETI debt of node *i* at slot *k*. The ETI debt of node *i* is

(33) $$\begin{array}{c} x_{i}\left(k\right)=\tau_{i}\left(k\right)-{\bar{h}}_{i} \end{array}$$

where ${\bar{h}}_{i}$ is the MATI value and $\tau_{i}\left(k\right)$ is the ETI at slot *k*.

When the scheduling scheme does not meet the ETI requirement ${\bar{h}}_{i}$, then the value of the ETI debt is positive. We define the positive part of the ETI debt, $x_{i}^{+}\left(k\right)=\max\left(x_{i}\left(k\right),0\right)$. As increasing debt $x_{i}^{+}\left(k\right)$ indicates to the scheduling scheme that node *i* needs more transmission slots to meet the MATI requirement.

Let $S_{k}=\left(x_{1}\left(k\right),\ldots ,x_{N}\left(k\right)\right)$ be a vector of network ETI dept states at slot *k*. Then, we define the Lyapunov Function by

(34) $$\begin{array}{c} V\left(S_{k}\right)=\frac{1}{2}\sum_{i=1}^{N}\left[{\left(x_{i}\left(k\right)\right)}^{2}+G{\left(x_{i}^{+}\left(k\right)\right)}^{2}\right] \end{array}$$

where *G* is a large positive value to emphasize the ETI constraints. Remark that $V(S_{k})$ is large when nodes have high ETI with respect to the requirement or positive ETI debt.

### Optimal Solution

To minimize the Lyapunov function, we introduce the Lyapunov drift

(35) $$\begin{array}{c} \Delta \left(S_{k}\right)=E\left[V\left(S_{k+1}\right)-V\left(S_{k}\right)|S_{k}\right] \end{array}$$

Note that the Lyapunov drift measures the change of the Lyapunov function over time slots. The Lyapunov-based scheduling scheme minimizes $V(S_{k})$ by reducing $\Delta (S_{k})$ in every slot *k*.

The high computation complexity of $V(S_{k})$ prevents us from deriving the exact form of $\Delta (S_{k})$. Hence, we derive the upper bound of $\Delta (S_{k})$ and apply it for the actual transmission scheduling scheme.

**Proposition** **3.** *The upper bound of* $\Delta (S_{k})$ *is*

(36) $$\begin{array}{c} \Delta \left(S_{k}\right)\leq -\sum_{i=1}^{N}E\left[u_{i}\left(k\right)|S_{k}\right]P_{i}\left(k\right)+Q\left(k\right) \end{array}$$

*where* $P_{i}\left(k\right)$ *and* Q(k) *are given by*

(37) $$\begin{array}{l} P_{i}\left(k\right)=p_{i}\tau_{i}\left(k\right)\left[x_{i}\left(k\right)+1+G\left(x_{i}^{+}\left(k\right)+1\right)\right]\; \end{array}$$

(38) $$\begin{array}{c} Q(k)=\sum_{i=1}^{N}\left[x_{i}\left(k\right)+\frac{\tau_{i}^{2}\left(k\right)}{2}+G\left(x_{i}^{+}\left(k\right)+\frac{\tau_{i}^{2}\left(k\right)}{2}\right)\right] \end{array}$$

**Proof.**  The proof of Proposition 3 is provided in Appendix C. □

We observe that both $P_{i}\left(k\right)$ and Q(k) are functions of the network state $S_{k}$ and setup parameters $\left(N,{\bar{h}}_{i},p_{i}\right)$. However, Q(k) of Equation (38) is not dependent on the scheduling decision $u_{i}\left(k\right)$. Hence, in each slot *k*, the centralized Lyapunov-based scheduling scheme selects the node with maximum value of $P_{i}\left(k\right)$ to minimize the upper bound of $\Delta (S_{k})$.

## 9. Performance Evaluation

In this section, we evaluate the performance of four transmission scheduling policies, namely ideal scheduling scheme, centralized random access scheme, distributed random access scheme, and centralized Lyapunov-based scheduling scheme. The ideal scheduling scheme optimizes the transmission schedule based on the entire packet loss sequences of each link. Hence, it gives the fundamental performance bounds even though it is not feasible to implement in practice. We considered a star network topology where *N* sensor nodes contend to send data packets to the controller. Each node has different sets of ${\bar{h}}_{i}$ and $p_{i}$. Note that each link *i* of the centralized scheduling approach has equal $p_{i}$ for both uplink and downlink. Let us denote the maximum MATI and minimum MATI of the network as $h_{\max}$ and $h_{\min}$, respectively. With a similar method, we define the maximum link reliability and minimum link reliability of the network $p_{\max}$ and $p_{\min}$, respectively. Then, the MATI and link reliability of node *i* are

(39) $$\begin{array}{c} {\bar{h}}_{i}=\frac{h_{\max}-h_{\min}}{N-1}\left(i-1\right)+h_{\min}\;\forall i\in \left\{1,\ldots ,N\right\}, \end{array}$$

(40) $$\begin{array}{c} p_{i}=\frac{p_{\max}-p_{\min}}{N-1}\left(i-1\right)+p_{\min}\;\forall i\in \left\{1,\ldots ,N\right\}. \end{array}$$

The lower are the values of ${\bar{h}}_{i}$ and $p_{i}$, the more challenging are the constraints. Hence, the lower node ID has more strict MATI requirement with worse link reliability. Each simulation ran $K=N\times 10^{8}$ time slots.

Both centralized schemes of static random access and Lyapunov-based schedule need the resource allocation message to assign the time slot to nodes. We assumed that both centralized schemes use one additional slot to transmit the resource allocation message. The centralized solutions consume two slots for the single data transmission while the distributed random access only requires one slot for the data transmission. Since the packet delay is fixed to 1 time slot, we mainly analyzed the performance of TI of different schemes.

**Figure 6:**
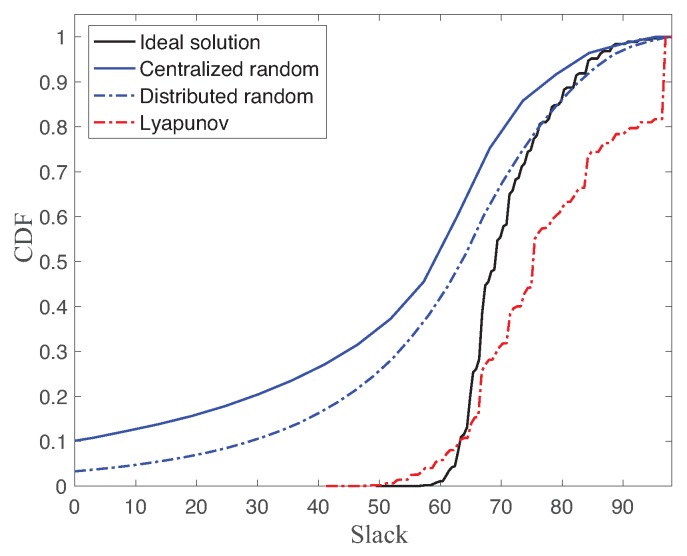
CDF of slack of ideal solution, centralized random access, distributed random access, and Lyapunov-based approach with N=8.

Figure 6 shows the cumulative density function (CDF) of slack of ideal solution, centralized random access, distributed random access, and Lyapunov-based approach with link reliability $p_{\min}=0.9,p_{\max}=1$, MATI requirement $h_{\min}=70,h_{\max}=100$, and number of nodes N=8. The slack is the difference between MATI and TI measurements. As a reminder, the minimum slack of TI with respect to MATI is the objective value of the proposed optimization problem. The higher is the slack, the better is the robustness. Hence, a lower CDF is better than a higher CDF for the stability guarantee. The Lyapunov-based scheduling scheme generally gives a lower CDF than other CDFs. However, our objective was to maximize the worst slack of the network. In fact, the worst slack value of the ideal solution is greater than the one of the Lyapunov-based approach in Figure 6. Hence, the ideal solution still provides the optimal solution of our proposed optimization problem in Equation (6) using the perfect knowledge of the packet loss sequences. The Lyapunov-based approach efficiently improves the robust performance based on the feedback slack information between TI and MATI of heterogeneous multiple control systems.

On the other hand, there is a significant gap between ideal solution with two randomized scheduling approaches, namely centralized random access and distributed random access. Note that the centralized random access does not rely on any feedback information of TI. The interesting observation is that the distributed random access generally performs better than the centralized one for N=8. Even though the distributed random access may incur the collisions with other transmissions, each data transmission requires only one time slot. While the centralized random access has no contention with other nodes, each data transmission consumes two time slots due to the resource allocation message. Since the collision probability is low for the small number of nodes, the distributed random access gives better robustness, as shown in Figure 6.

Another interesting point is the different percentile values between four schemes. While the four schemes have similar 50th percentile values, the percentile difference between different approaches increases as the percentage decreases. It means the worst case performance of the proposed approaches is significantly different.

**Figure 7:**
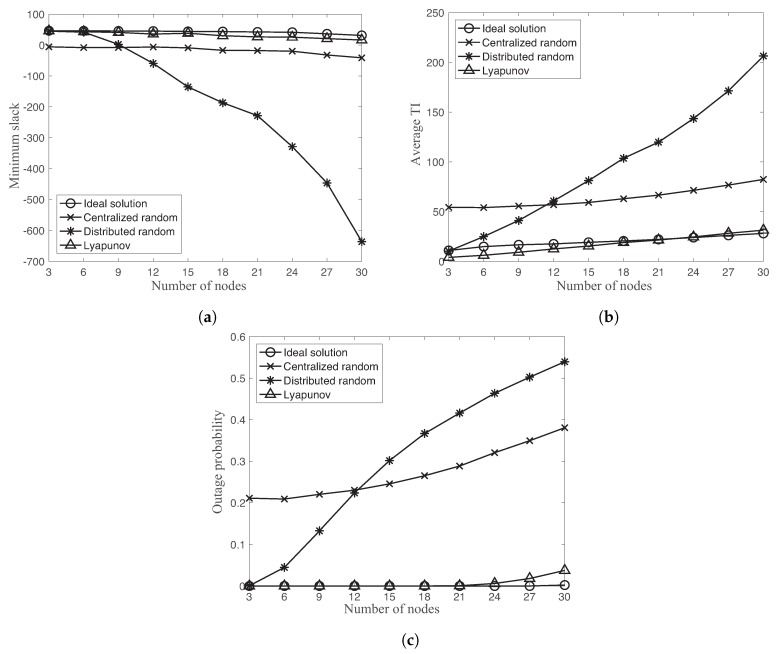
Minimum slack, average TI, and outage probability of ideal solution, centralized random access, distributed random access, and Lyapunov-based approach as a function of different number of nodes N=3,…,30: (**a**) minimum slack vs. number of nodes; (**b**) average TI vs. number of nodes; and (**c**) outage probability vs. number of nodes.

Figure 7 shows the minimum slack, average TI, and outage probability of four different schemes as a function of different number of nodes N=3,…,30 with link reliability $p_{\min}=0.9,p_{\max}=1$, and MATI requirement $h_{\min}=50,h_{\max}=100$. The outage probability is defined as the probability that the TI value is greater than MATI, ${\bar{h}}_{i}$.

Let us first compare the performance between ideal solution and Lyapunov-based approach. In Figure 7a,c, the ideal solution provides the lower outage probability and higher minimum slack than the one of the Lyapunov-based approach. However, the average TI of the ideal solution is slightly higher than the one of Lyapunov-based approach in Figure 7b. As a reminder, the objective of the proposed optimization problem is to maximize the minimum TI slack with respect to MATI requirements instead of minimizing average TI value of the network. The average TI is not explicitly considered in the optimization problem.

While the Lyapunov-based approach is comparable with the ideal solution, both random accesses show significantly different behaviors. In Figure 7, the centralized random access has an almost constant gap of the minimum slack, average TI, and outage probability with the Lyapunov-based approach over different number of nodes. On the other hand, the distributed random access significantly degrades these performance metrics as increasing the number of nodes. By comparing two random accesses, the distributed random approach provides better performance than the centralized random access for the small number of nodes N≤9. There is the fundamental stability limits of the distributed random access approach due to the increasing collision probability dependent on the number of nodes [38].

**Figure 8:**
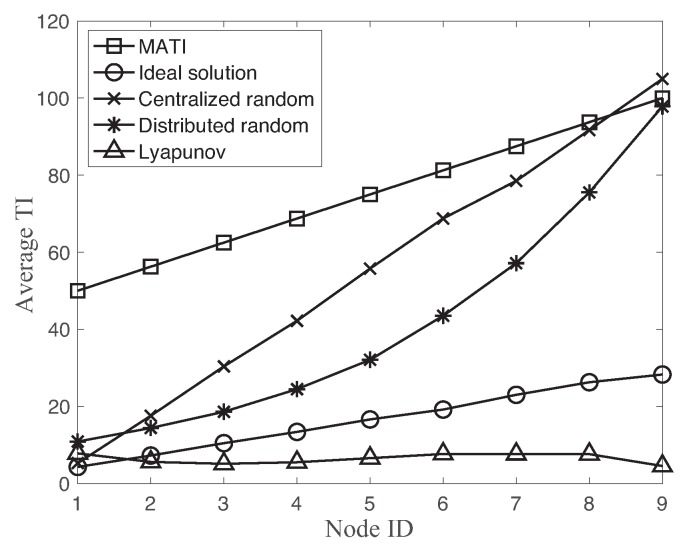
MATI requirement and average TI of each node using different transmission scheduling schemes with N=9.

Figure 8 shows the MATI requirement and the average TI of each node using different transmission scheduling schemes with N=9. As a reminder, the MATI requirement and link reliability become more strict as decreasing node ID of the network. We observe that the average TI of the ideal solution is decreasing as the MATI requirement becomes more strict for the lower node ID. However, the Lyapunov-based approach is quite flat over different MATI requirements with respect to the ideal solution. The node ID 1 provides the minimum slack of both ideal solution and Lyapunov-based approach of the network. This is the main reason of the greater minimum slack of the ideal solution at the cost of the higher average TI compared to the Lyapunov-based approach in Figure 7.

On the other hand, both random accesses significantly increase the average TI as the MATI requirement becomes relaxed for the higher node ID. In both random accesses, the slack between MATI and average TI is decreasing as the node ID increases. In fact, the minimum slack value of both random accesses occurs at node ID 9.

**Figure 9:**
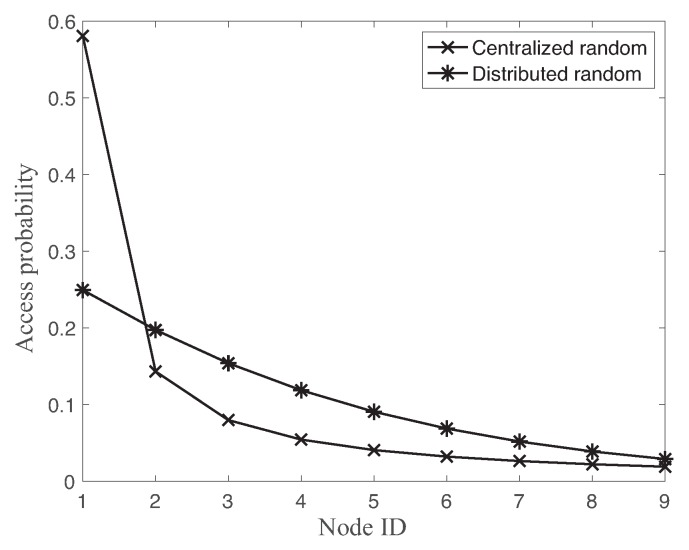
Access probability of each node using centralized random access and distributed random access schemes with N=9.

While the centralized random access supports the stationary optimal resource allocation, its operation requires the additional slots for the resource allocation message. In fact, each successful data transmission of the centralized approaches needs both successful transmissions of the resource allocation message and data packet. Hence, the performance of the random access is a complex function of number of nodes, link reliability, and operational overhead. Figure 9 compares the channel access probabilities of each node using both centralized random access and distributed random access with N=9. Both solutions of random accesses assign the higher access probability to the higher priority node ID, i.e., lower node ID. While the channel access probability of distributed random access is mush smoother for different nodes, the centralized random access approach sets the very high channel access probability 0.58 for the most demanded node ID 1. In fact, both random accesses show the over-allocation of slot resources to the most demanded node as shown in Figure 8.

## 10. Conclusions

In this paper, we consider the transmission scheduling schemes of industrial wireless sensors for heterogeneous multiple control systems over unreliable wireless channels. We first discuss the fundamental tradeoffs of the TI and the packet delay of wireless networks for the control stability. Based on the fundamental observation, we formulate the constrained optimization problem of maximizing the minimum slack of the TI with respect to the maximum allowable requirement of all network nodes. We propose three low-complex transmission scheduling schemes, namely centralized stationary random access, distributed random access, and centralized Lyapunov-based scheduling scheme, to solve the proposed optimization problem. The simulation results show that the centralized Lyapunov-based scheduling approach provides robust performance closer that is to the ideal solution by using the feedback state information. Furthermore, the distributed random access is another good candidate of the transmission scheduling for the small number of control loops.

The practical validation of the proposed scheduling scheme is critical in the context of industrial setup [3]. Future investigations include the practical implementation of different scheduling schemes using Zolertia sensors [43] based on the specifications of the IEEE 802.15.4 standard. Furthermore, we are planning to extend the proposed framework to balance the control cost and the energy efficiency while considering the additional constraints using the energy harvesting techniques [44].

## Appendix A. Proof of Proposition 1

**Proof.** In each slot *k*, the controller successfully receives a packet if the node is scheduled with probability $\alpha_{i}$ and the link condition is good with probability $p_{i}$. Hence, the probability of successful transmission of node *i* is $p_{i}\alpha_{i}$. Let us denote $h_{i}$ as the TI of successful received packet of node *i*. Then, the sum of ETI is

$$\begin{array}{c} \sum_{k=1}^{K}\tau_{i}\left(k\right)=1+2+\ldots +h_{i}=\frac{h_{i}^{2}+h_{i}}{2} \end{array}$$

Note that $h_{i}$ follows $P\left[h_{i}=j\right]=p_{i}\alpha_{i}{\left(1-p_{i}\alpha_{i}\right)}^{j-1},\forall j\in Z^{+}$ since it is independent and identically distributed. By using renewal theory, the expected ETI of node *i* becomes

(A1) $$\begin{array}{c} \frac{1}{K}\sum_{k=1}^{K}E\left[\tau_{i}\left(k\right)\right]=\frac{1}{E[h_{i}]}\left(\frac{E\left[h_{i}^{2}\right]+E\left[h_{i}\right]}{2}\right) \end{array}$$

Hence, the long-term expected ETI of node *i* is

(A2) $$\begin{array}{c} \lim_{K\to \infty }\frac{1}{K}\sum_{k=1}^{K}E\left[\tau_{i}\left(k\right)\right]=\frac{E[h_{i}^{2}]}{2E[h_{i}]}+\frac{1}{2}=\frac{1}{p_{i}\alpha_{i}} \end{array}$$

due to its generalization for renewal-reward processes [45]. □

## Appendix B. Proof of Proposition 2

**Proof.** Since the logarithmic function is strictly increasing, each ETI constraint of Equation (26b) is equivalently rewritten as $\nu \leq \log a_{i}+\log\beta_{i}+\sum_{j\in C_{i}}\log\left(1-\beta_{j}\right),\forall i\in \left\{1,\ldots ,N\right\}$ where ν=logψ. Hence, the constraint becomes

(A3) $$\begin{array}{c} \nu -\log a_{i}-\log\beta_{i}-\sum_{j\in C_{i}}\log\left(1-\beta_{j}\right)\leq 0\;. \end{array}$$

Note that each constraint of Equation (A3) shows a convex set of (β,ν) with the monotonically increasing logarithmic function. Hence, the optimization problem of Equation (26) is reformulated as the following convex problem

(A4a) $$\begin{array}{c} \max\;\nu \; \end{array}$$

(A4b) $$\begin{array}{c} s.t.\;\nu \leq \log a_{i}+\log\beta_{i}+\sum_{j\in C_{i}}\log\left(1-\beta_{j}\right)\;. \end{array}$$

Since we assume the fully connected graph of the single hop network, all $\nu_{i}$ must have equal value, namely $\nu_{i}=\nu_{j}$ for all i∈{1,…,N} and $j\in C_{i}$. By assuming $a_{i}\beta_{i}\prod_{j\in C_{i}}\left(1-\beta_{j}\right)\leq 1$, we have ν≤0. Hence, maximizing ν is equivalent to minimize $\nu^{2}$ in the optimization problem. Therefore, it is possible to rewrite the non-convex optimization problem in Equation (26) to the convex problem in Equation (27). □

## Appendix C. Proof of Proposition 3

**Proof.** In this appendix, we obtain the upper bound of the Lyapunov drift $\Delta (S_{k})$ of Equation (36). Let us consider the network state $S_{k}$, the Lyapunov function $V(S_{k})$ in Equation (34), and the Lyapunov drift $\Delta (S_{k})$ in Equation (35). After plugging Equation (34) into Equation (35), we obtain

(A5) $$\begin{array}{c} \Delta (S_{k})=\frac{1}{2}\sum_{i}^{N}E\left[x_{i}^{2}\left(k+1\right)-x_{i}^{2}\left(k\right)|S_{k}\right]+\frac{G}{2}\sum_{i}^{N}E\left[{\left(x_{i}^{+}\left(k+1\right)\right)}^{2}-{\left(x_{i}^{+}\left(k\right)\right)}^{2}|S_{k}\right] \end{array}$$

In Equation (A5), we derive the expressions of ${\left(x_{i}^{+}\left(k+1\right)\right)}^{2}-{\left(x_{i}^{+}\left(k\right)\right)}^{2}$ and $x_{i}^{2}\left(k+1\right)-x_{i}^{2}\left(k\right)$. The ETI update of Equation (3) is rewritten as

(A6) $$\begin{array}{c} \tau_{i}\left(k+1\right)=\tau_{i}\left(k\right)\left(1-d_{i}\left(k\right)\right)+1 \end{array}$$

By combining Equations (33) and (A6), the iteration of the ETI debt is

(A7) $$\begin{array}{c} x_{i}\left(k+1\right)=x_{i}\left(k\right)-\tau_{i}\left(k\right)d_{i}\left(k\right)+1, \end{array}$$

where $x_{i}\left(1\right)=0$. Hence, ${\left(x_{i}^{+}\left(k+1\right)\right)}^{2}$ becomes

(A8) $$\begin{array}{c} {\left(x_{i}^{+}\left(k+1\right)\right)}^{2}={\left[\max\left(x_{i}\left(k\right)-\tau_{i}\left(k\right)d_{i}\left(k\right)+1,0\right)\right]}^{2} \end{array}$$

(A9) $$\begin{array}{l} \leq {\left[\max\left(x_{i}^{+}\left(k\right),0\right)\right]}^{2}\; \end{array}$$

(A10) $$\begin{array}{l} \leq {\left(x_{i}^{+}\left(k\right)\right)}^{2}\; \end{array}$$

Equation (A10) is rewritten as

(A11) $$\begin{array}{c} {\left(x_{i}^{+}\left(k+1\right)\right)}^{2}-{\left(x_{i}^{+}\left(k\right)\right)}^{2}\leq -2\tau_{i}\left(k\right)\left(x_{i}^{+}\left(k\right)+1\right)d_{i}\left(k\right)+2x_{i}^{+}\left(k\right)+\tau_{i}^{2}\left(k\right)\;. \end{array}$$

By taking the expectation of Equation (A11), we compute the upper bound

(A12) $$\begin{array}{c} E\left[{\left(x_{i}^{+}\left(k+1\right)\right)}^{2}-{\left(x_{i}^{+}\left(k\right)\right)}^{2}|S_{k}\right]\leq -2\tau_{i}\left(k\right)\left(x_{i}^{+}\left(k\right)+1\right)p_{i}E\left(u_{i}\left(k\right)|S_{k}\right)+2x_{i}^{+}\left(k\right)+\tau_{i}^{2}\left(k\right) \end{array}$$

With a similar method, we get

(A13) $$\begin{array}{c} E\left[{\left(x_{i}\left(k+1\right)\right)}^{2}-{\left(x_{i}\left(k\right)\right)}^{2}|S_{k}\right]\leq -2\tau_{i}\left(k\right)\left(x_{i}\left(k\right)+1\right)p_{i}E\left[u_{i}\left(k\right)|S_{k}\right]+2x_{i}\left(k\right)+\tau_{i}^{2}\left(k\right) \end{array}$$

After substituting Equations (A12) and (A13) into the Lyapunov drift of Equation (A5), we obtain the upper bound of $\Delta (S_{k})$ as Equations (36)–(38). □
